# Treating severe wounds in pediatrics with medical grade honey: A case series

**DOI:** 10.1002/ccr3.2691

**Published:** 2020-01-31

**Authors:** Eleftherios Smaropoulos, Niels A. J. Cremers

**Affiliations:** ^1^ Department of Pediatrics Aristotle University of Thessaloniki Thessaloniki Greece; ^2^ Department of Pediatrics St. Luke Private Clinic Thessaloniki Greece; ^3^ Triticum Exploitatie BV Maastricht The Netherlands

**Keywords:** burns, injuries, medical grade honey, pediatrics, trauma, wounds

## Abstract

Medical grade honey (MGH) has antimicrobial and pro‐healing properties. We here demonstrate that MGH is an easily applicable, safe, and cost‐effective approach for severe wounds. The use of MGH should more often be considered to treat all kinds of pediatric wounds.

## Graphical abstract

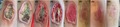

## 1 INTRODUCTION

Honey has been used throughout history for the treatment of wounds. Medical grade honey (MGH) made a resurgence upon the development of antibiotic resistance; however, the use of MGH in pediatric patients remains infrequent. Here, we present a case series showing the ease, safety, and efficacy of MGH in severe injuries in pediatric patients. In this prospective observational study, five pediatric patients with different wounds were included, one extravasation‐induced injury, one hemangioma, one coccyx ulcer, and two thermal burns. All wounds were treated in the same manner, via monotherapy with daily MGH application and close monitoring. All wounds showed rapid wound progression, as demonstrated by the fast granulation tissue formation and epithelialization, and this was independent of the location or the severity of the wound. Slough and necrotic tissue were effectively debrided. Application of MGH was easy and did not cause any pain to the patients. MGH possesses pro‐healing and antimicrobial activity, both contributing to enhanced wound repair. In pediatric patients, MGH therapy can be an easy and cost‐friendly approach to control infections and effectively heal severe wounds. The use of MGH should more often be considered to treat pediatric wounds.

Children are susceptible to injuries and wounds that require medical attention. Despite being treated in a controlled hospital setting, wounds are at risk of nosocomial infections. Therefore, there is an urgent need to improve wound healing protocols that prevent infections and avoid unnecessary use of antibiotics.

Honey is a natural substance used for wound healing since ancient times.^1^, ^2^, ^3^ The use of antibiotics made the use of honey for wound healing less frequent. However, the development of antibiotic resistance led to the resurgence of medical grade honey (MGH).^4^ MGH fulfills several criteria to guarantee its quality and make its use safe.^4^ MGH is collected in an eco‐friendly environment in the absence of pollutants, including herbicides, pesticides, and heavy metals.^4^ Besides, MGH is sterilized using gamma irradiation to kill spores of *Clostridium botulinum*, without inactivating the enzymes that are needed for its antimicrobial and wound healing activities.^4^, ^5^

The application of MGH to a wound can prevent infection and invasion of pathogens by forming a physical shield.^6^ Additionally, MGH possesses multiple characteristics that orchestrate its antimicrobial activity, and therefore also has a limited risk for the development of antimicrobial resistance.^3^ The acidic pH, the osmotic effect caused by the high sugar content, and the release of hydrogen peroxide after the enzymatic breakdown of sugar act antimicrobial.^1^, ^6^, ^7^, ^8^ Other molecules that can be present in honey and have antibacterial effects are phenolic compounds, flavonoids, methylglyoxal, and Bee defencin‐1.^1^, ^3^, ^9^, ^10^, ^11^ Together, these characteristics make MGH effective against a broad range of microorganisms, including those resistant against antibiotics.^3^ MGH can serve as an alternative or complementary treatment for antibiotics in local infections, but also its prophylactic use has been demonstrated.^12^ Moreover, MGH has pro‐healing effects, keeps the wound moisturized, promotes autolytic debridement, has anti‐inflammatory and antioxidative effects, and induces angiogenesis and reepithelialization.^1^, ^2^, ^13^, ^14^, ^15^ Honey also contains protective molecules such as phenolic compounds and flavonoids that further enhance wound healing.^16^, ^17^

It has been demonstrated that MGH can safely and effectively improve tissue regeneration in a wide variety of wounds, covering the whole range of wound types, from black necrotic wounds to infected yellow wounds and superficial red wounds. Examples of wounds that can be treated are traumatic injuries, burns, hematomas, pressure ulcers, diabetic foot ulcers, full and partial thickness wounds, lacerations, and skin tears.^1^, ^2^, ^14^, ^15^, ^18^, ^19^ To get a generalized idea of the effects of MGH in a pediatric setting, we now present five cases with severe wounds that frequently occur. Hereby, we aim to illustrate that MGH is a safe, easy, and cost‐effective approach to control infections and to enhance wound healing in a pediatric patient population.

## 2 METHODS

### 2.1 Materials

Wounds were treated with L‐Mesitran ointment (provided by Triticum, Maastricht, the Netherlands). This honey‐based wound care product contains 48% medical grade honey,^4^ hypoallergenic lanolin, vitamin C, vitamin E, zinc oxide, and essential oils. Several studies have demonstrated that the supplements in L‐Mesitran enhance the antimicrobial properties of its raw honey^20^, ^21^ and is more potent than Manuka‐based Medihoney.^22^

### 2.2 Subjects

Five pediatric patients with age between 3 weeks and 9 years, admitted to the hospital in Thessaloniki, Greece, and presenting with severe wounds, were evaluated in our prospective observational case series. The wounds included were an extravasation‐induced injury, a hemangioma, a coccyx ulcer, and two patients with thermal burns. The study was carried out in accordance with relevant institutional and national guidelines, and the protocol was approved by the Ethics committee of St Luke's hospital (Panorama, Thessaloniki, Greece). The principles of the World Medical Association's Declaration of Helsinki were followed. The patients’ legal representatives gave written informed consent to participate in the study and publication of the data.

### 2.3 Treatment

All cases, with the exception of case 5, received equal treatment; a formulation of medical grade honey (MGH) (provided by Triticum) was used as monotherapy.^4^ The wound area was cleaned using saline solution (0.9%), and MGH was subsequently applied locally to the wound, which was repeated daily. MGH was applied superficial to the wound in a thin layer (1‐2 mm) to cover the wound, or as a thick layer (up to 10‐12 mm) in the deeper wounds (cases 2 and 3). All wounds received regular simple sterile gauze as secondary dressings. The wounds treated on inpatient basis (cases 1, 3, and 5) were closely monitored by the nurses and doctors, and MGH was freshly applied every day. Cases 2 and 4 were treated on an outpatient basis, and strict wound care guidelines were provided to the parents. From the latter, wound pictures were daily sent to the physician and patients were appointed for weekly consultation. The patient with severe burns in case 5 received partially different treatment: The burn on the leg was treated with MGH while the burns on the rest of the body were treated with povidone iodine and skin grafting. No sedatives, pain medications, antibiotics, or other topical therapies were given (except as mentioned for case 5) because none of the patients presented any discomfort or pain. No fever was measured during the treatment period, except for case 5, with a temperature of 37.5‐38°C the first day of the incident. There was no clear infection evidence in any of the patients, and CRP and WBC levels were within the normal range; the only signs were necrotic tissue and slough (cases 1‐3). When heavy slough was present (cases 1 and 2), this was carefully removed by scrubbing with saline‐wetted gauze before the application of new MGH.

## 3 RESULTS

### 3.1 Case 1: Large necrotic extravasation wound

Patients that are hospitalized and need frequent infusions, for example, patients that are malnourished, are at risk to develop extravasation‐induced injuries. Extravasation is the accidental leakage of fluid, such as medication or nutrients, into the surrounding tissue during infusion. Subsequently, this can lead to skin and soft tissue damage and result in severe ischemia, infection, and necrosis.^23^, ^24^

**Figure 1:**
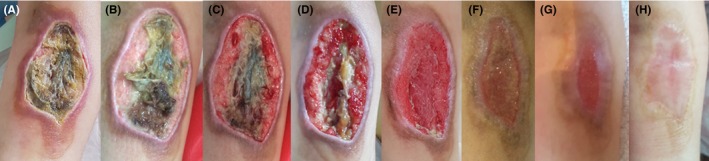
Large necrotic extravasation wound in a 9‐year‐old girl. A, The appearance of the necrotic wound at the start of the MGH treatment (day 0). B, After 5 d, granulation tissue is formed, and the necrotic tissue in the center is reduced, which was further removed surgically (C). D, Wound at day 14. E, Necrotic tissue disappeared after 3 wk of MGH therapy. F, After 30 d, the wound progressively healed. G, The result after 56 d. H, Follow‐up after 5 additional weeks

A 9‐year‐old girl, with quadriplegia and severe brain damage as a consequence of a viral infection, was hospitalized for prolonged periods in the pediatric intensive care unit (PICU) because of intestinal septic episodes. The extravasation of drugs and intravenous solutions stayed unnoticed until the patient presented a stage IV ulcer with severe tissue necrosis of her forearm, covering almost the complete width (Figure 1A). The necrotic epidermis was surgically debrided, and MGH monotherapy was started to further enhance autolytic debridement and healing. At day 5, granulation tissue was formed, while there was a clear reduction in necrotic tissue in the center of the lesion (Figure 1B), which was further removed surgically, leaving the tissue looking healthier (Figure 1C). Two weeks after MGH therapy started, the wound shows the formation of granulation tissue from the borders to the center and further reduction of necrotic tissue (Figure 1D). At week 3, the necrotic tissue was completely absent, and the wound decreased in size (Figure 1E). During week 5, the wound gradually epithelialized with healthy granulation tissue and new formation of blood vessels under the skin indicated by the bright color (Figure 1F). The wound properly healed after 56 days of MGH therapy (Figure 1G). Follow‐up after 5 more weeks showed a good cosmetic result (Figure 1H). MGH application was easy, and the patient confirmed a lack of pain or discomfort during or due to the wound care.

### 3.2 Case 2: Ulcerated hemangioma

Hemangiomas are benign vascular tumors that can be present at birth (congenital) or occur during childhood (infantile) with an incidence of 3%‐10% in all infants and are frequenter experienced in females and preterm infants.^25^ Most uncomplicated hemangiomas do not need treatment. However, ulceration occurs in up to 15% of the cases leading to pain and risk of infection and scarring, and an intervention is therefore inevitable.^25^ Treatments are often restricted to wound management, conservative therapies, laser treatment, or surgery. Here, we present a case of an ulcerated hemangioma that is treated with MGH.

**Figure 2:**
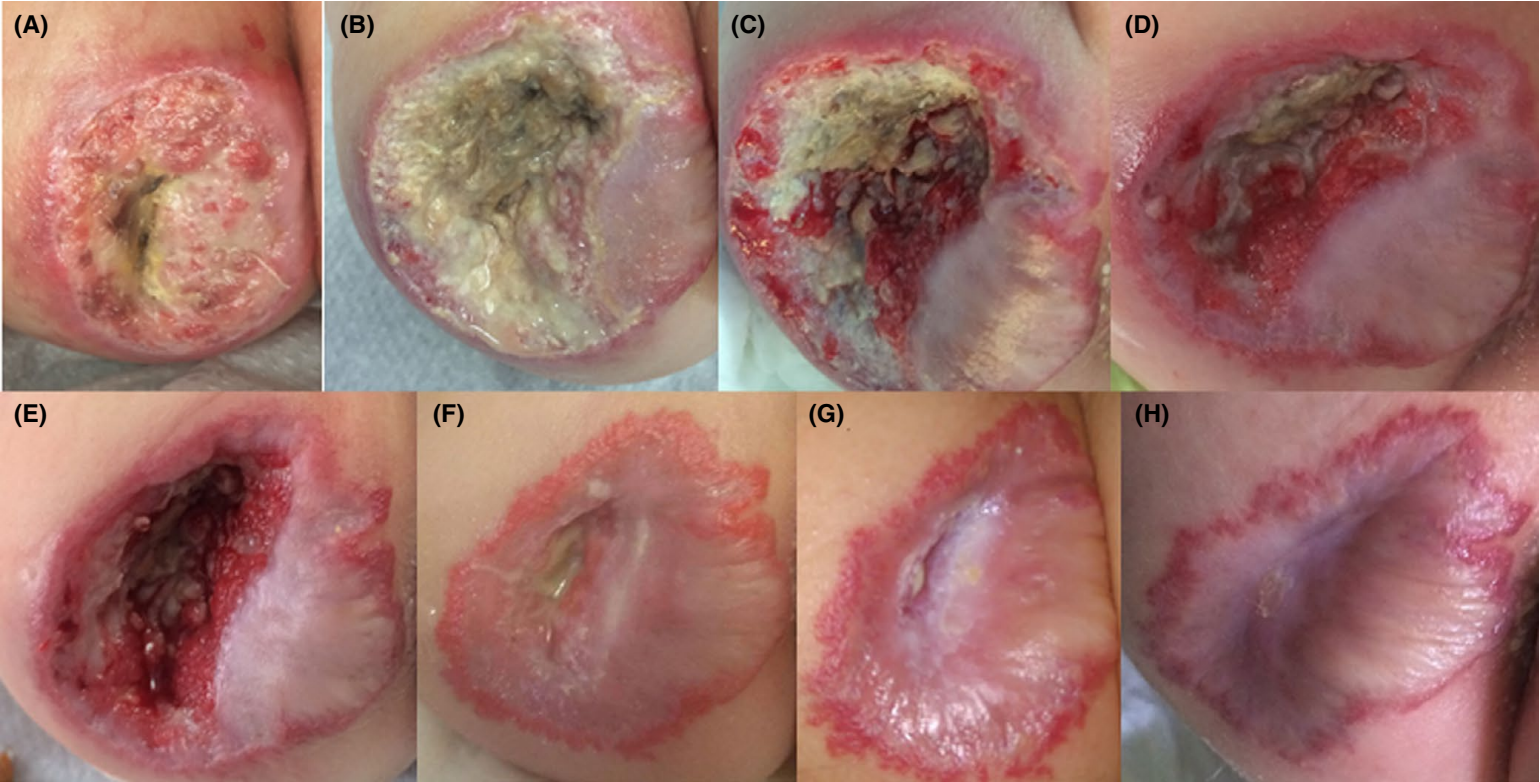
Ulcerated hemangioma in a 7‐mo‐old baby. A, Start of MGH treatment (day 0). B, Concave slough wound 2 wk after the start of therapy. C, Sloughy wound after 21 d, which was subsequently removed (D). E, After 28 days, slough was again removed, leaving a healthier and viable looking tissue with room to form granulation tissue. F, Progressive wound healing with almost complete epithelialization at day 40. G, The wound was almost closed at day 49 with the borderline of the preexisting hemangioma remaining. H, Complete healing and nice result after 69 d

A 7‐month‐old female toddler born with a congenital hemangioma presented with a large mixed ulcerated hemangioma on the right thigh. The stage IV ulcer was superficial and deep and had a size of about 6 × 7 cm. Until presentation, the vascular supply of the mass was compromised and showed clear signs of necrosis, while the parents waited for the evolution of the skin necrosis, leaving the ulcer untreated. At this time, laser and surgical therapy were not feasible, and monotherapy with MGH seemed the best option and was started (Figure 2A). The ulcer was almost covering the complete right thigh and exhibited a concave, sloughy state with a lot of necrotic tissue and malodor. The vasculature was strongly compromised by the large size and depth of the ulcer. After 14 days of MGH therapy and surgical debridement, a large sloughy concave wound lacking subcutaneous tissue was observed (Figure 2B). On day 21, some epithelialization was visible at the edges; however, there was still a lot of slough present (Figure 2C), which was subsequently removed, leading to a healthier tissue color and making space for the formation of granulation tissue (Figure 2D). After the removal of the slough at day 28, although the wound was still quite deep, the tissue looked viable and clear reepithelialization was visible (Figure 2E). After 40 days, the wound progressively healed and in relation to the initial size, only a small part of the wound was still open (Figure 2F). At day 49, the wound was almost completely healed, while the border of the preexisting hemangioma still held a different brighter red color than the rest of the skin (Figure 2G). Only 69 days after MGH therapy, the ulcerated hemangioma was completely healed, with full epithelization and a decrease in the circumference of the lesion (Figure 2H). Considering the original size and depth of the wound and the severely compromised vasculature, a nice result was achieved. During the visitations by the treating physician and according to the parents, MGH application was easy and did not cause any signs of pain or discomfort (based on vocal or verbal expression, facial expression, movements, postures, interaction with the environment).

### 3.3 Case 3: Bedsore coccyx

Pressure injuries are a challenging problem in the care of medically complex children, and therapies are mainly based on adult data, while there are considerable differences in body composition.^26^ Pressure injuries arise as a result of pressure and/ or shear and are often located over a bony prominence or related to a medical device. Risk factors include limited mobility, inability to reposition, loss of sensation, nutritional deficiency, cognitive impairment, or other pressure ulcers.^26^

**Figure 3:**
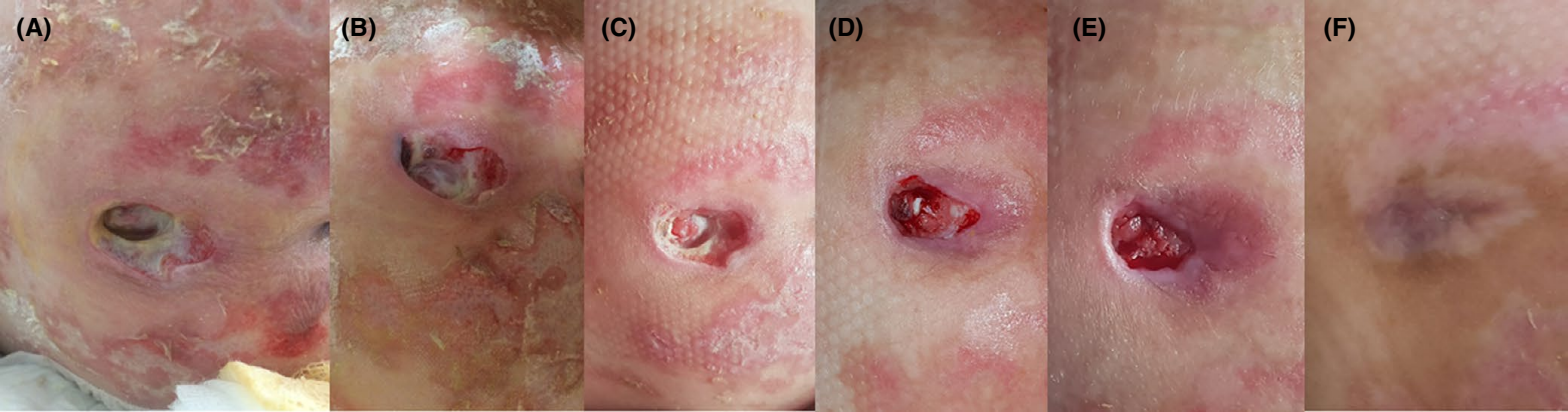
Bedsore coccyx in mobile impaired 8‐year‐old girl. A, Wound at first presentation and start of MGH treatment (day 0). B, Wound at day two of therapy. C, Less inflammation at day 8. D, Wound progressively healed at day 24. E, Wound after 32 d of MGH therapy. F, Follow‐up after 6 additional months

An 8‐year‐old female child was admitted to the hospital with respiratory problems and presented with severe bedsore ulcer and inflammation of her buttocks. The patient suffered for 4 years from paraplegia and severe impairment in motility of the upper body due to viral encephalitis In addition, the child had a tracheostoma and suffered from chronic intestinal dysmotility for which she was previously received surgery. At consultation, the coccyx ulcer was at stage IV (Figure 3A) and MGH therapy was directly started (day 0). To offload the pressure, the child was placed every 4 hours in a different lateral position and an inflatable rubber ring was used to keep the coccyx up from the bed surface. MGH therapy was started upon hospital admittance, and after 2 days, the slough was less pronounced, while hemostasis became visible and epithelialization from the edges started (Figure 3B). At day 8, the wound decreased in size and the tissue was less inflamed and appeared healthier (Figure 3C). At day 24, the slough completely disappeared while the wound progressively healed, indicated by the reduced wound size, covering part of the periosteum, a more vascularized wound bed, granulation tissue formation, and clear epithelialization (Figure 3D). After 32 days, the wound became smaller and more superficial, and the periosteum was completely covered (Figure 3E). MGH application was easy. As the patient suffered from paraplegia, no pain or discomfort was experienced. Follow‐up after 6 months showed a good cosmetic outcome (Figure 3F).

## 4 TWO BURN CASES

### 4.1 Case 4: Local burn of the wrist

Burn injuries in children continue to be a major epidemiologic problem around the globe and affect millions of children worldwide.^27^ Burns can be classified by their percentile coverage of the total body surface area (%TBSA) and depth of the injury. While the mortality rate following major burns at highly specialized centers is <3%, new therapies that prevent and treat possible infections while inducing wound repair are warranted. Next, we describe two cases with burns.

**Figure 4:**
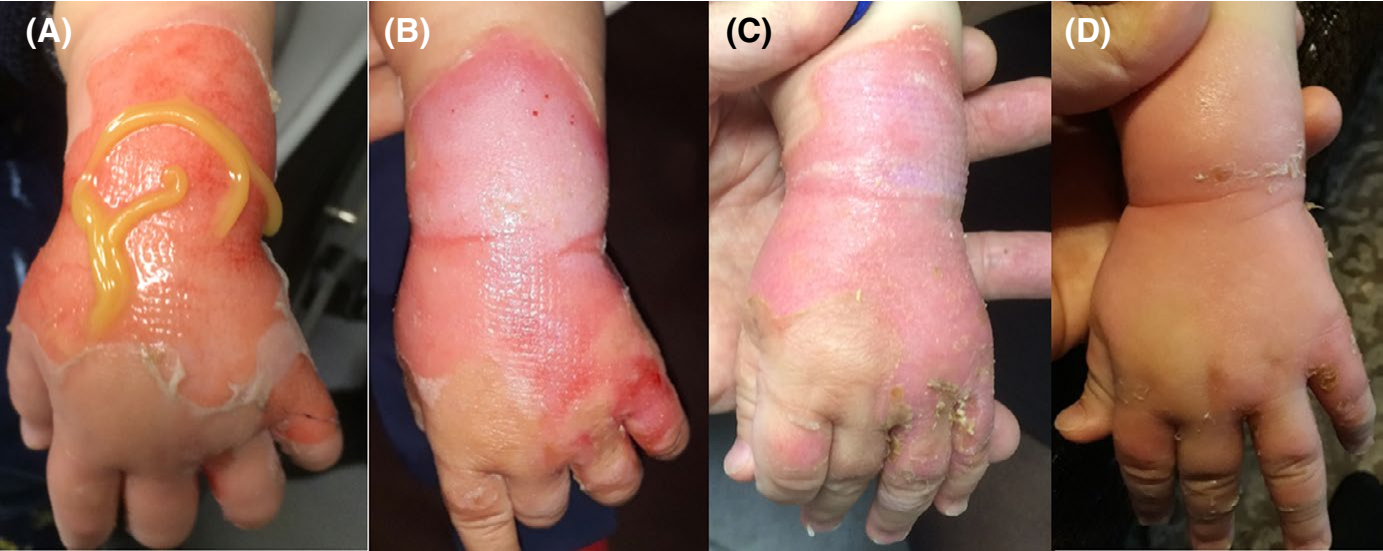
Burned skin around the wrist in an 8‐month‐old infant (A‐D). A, Start of MGH therapy (day 0). B, Burn on day 4. C, Reepithelialized wound after 7 d. D, Cosmetic and functional restoration after 12 d

An 8‐month‐old male infant suffered from a second‐degree (partial thickness) burn trauma of his left hand by exposure to boiling coffee. Treatment with MGH monotherapy was directly started (Figure 4A). After 4 days, the burn progressively healed as shown by the change in color from red into pink with the absence of inflammation (Figure 4B). After 7 days, the wound was completely epithelialized (Figure 4C), and cosmetic and functional restoration after 12 days (Figure 4D). According to the parents, which took care of the wounds, MGH was easy to apply and did not cause any visible pain or discomfort to their baby.

### 4.2 Case 5: Severe burn of the back and leg

**Figure 5:**
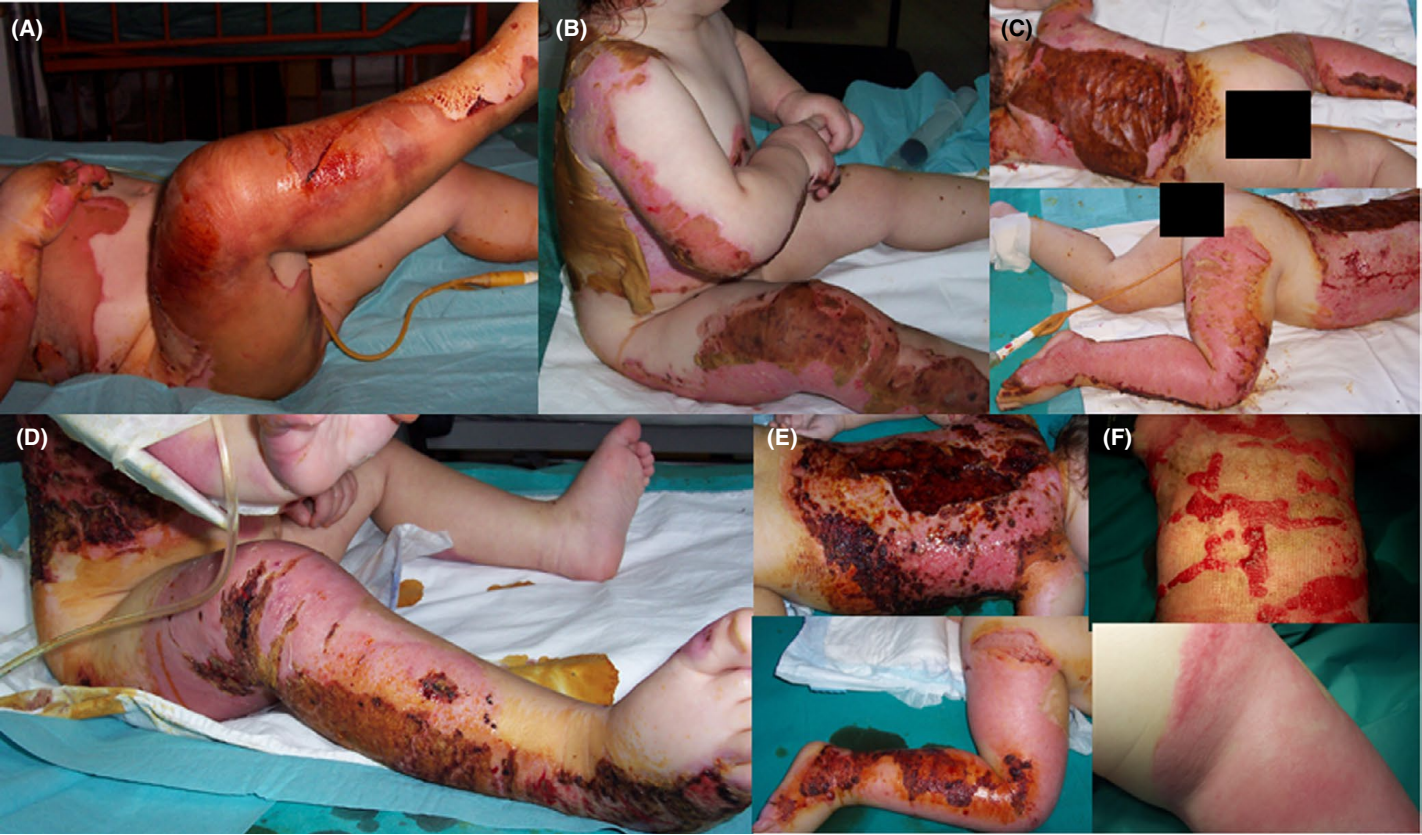
Severe burn wound in 18‐mo‐old girl. A, Toddler with severe burns located on a large part of her body (day 0). B, Detached necrotic skin on the back, and crust on the knee (day 5). C, Top: severe eschar on the back. Bottom: progressive healing of the leg (day 8). D, The crust on the knee healed by reepithelialization while the wound on the back slowly progressed (day 13). E, Eschar on the back was still present (top) while the healing in the leg was evident (bottom) at day 18. F, Wound on the back after receiving skin grafts, including from the healed right leg (top) and acceptable result of the leg (bottom) at day 94 of MGH therapy

An 18‐month‐old female toddler got burned from boiling water by a kettle falling on top of her. She suffered severe third‐degree burns (full thickness) on her hand, limbs, neck, back, and the front thoracic area covering 35% of her TBSA (Figure 5A). Her general health condition was critical due to the burns, pyrexia, and metabolic and electrolytic disorders. Pain medication (Zantac [Ranitidine], Human ALB, Perfalgan, and Paracetamol) and antibiotics (Zetagal [Cefuroxime Sodium], Solvetan [Ceftazidime] and Briklin [Amikacin]) were given. With permission of the child's parents, only the right leg was treated with a combination of L‐Mesitran Ointment and Hydro, while the rest of the burns received standard of care, comprising of initial application of povidone iodine and subsequent skin grafting (day 0). The wound on the right leg was of similar severity to the wounds on the rest of the body, all third‐degree full‐thickness burns.

After 5 days, the thigh already showed some healthy‐looking pink color, while the more severe burned location at the knee showed some crust formation, and the back of the child showed some detached necrotic tissue (Figure 5B). Eight days after MGH therapy, there was still severe eschar present on the back, while the healing at the MGH‐treated leg clearly progressed (Figure 5C, top, and bottom, respectively). At day 13, the wound at the knee showed proper reepithelialization, while the wound of the back slowly progressed (Figure 5D). After 18 days, necrotic eschar was still severely present on the back (Figure 5E, top), in contrast to the right leg in which healing of the right leg was evident at this time point (Figure 5E, bottom). Surgical debridement of the wound on the back in combination with split‐thickness autografts application was performed, resulting in complete wound coverage at day 94 (Figure 5F, top). At this time point, the leg showed an acceptable result (Figure 5F, bottom). Because of the limited available donor sites and since the right leg healed so well, the healed skin of the leg was used as donor area to advance the wound healing of the back. The grafted area was also effectively and easily treated with MGH. No pain or discomfort was observed during MGH therapy. Povidone iodine solution was a little painful upon application and created by time a thick hard necrotic tissue on the wound surface that had to be removed by scrubbing every 5‐7 days. To make the necrotic tissue easier to remove, pharmaceutical Vaseline was applied to the surface 4 hours prior to scrubbing. The child had to take pain killers for that procedure. On the contrary, with MGH, no signs of pain or discomfort were noticed. This is the only case in which antibiotics were given.

## 5 DISCUSSION

Pediatric patients are at high risk of injuries and wounds, such as burns, trauma, or surgery. A slow wound repair with intensive therapies can be a medical and economic burden for young pediatric patients and their parents. Therefore, broad and easily applicable, cost‐effective approaches are warranted to improve wound care. The use of MGH is a promising strategy to fight infections and improve healing of wounds of various origin and severity.^28^, ^29^ No infections arose during the treatment, possibly because MGH protected the wound from invading pathogens and possessing strong antimicrobial activity. MGH forms an interesting, natural, and efficient alternative for antibiotics. Implementation of MGH resulted in full wound healing in all presented cases.

Other studies also showed that MGH enhances wound healing. In patients that were treated for partial‐thickness facial burns, the healing time was better than what would have been expected with standard treatment, and the cost‐effectivity of honey was supported.^30^ Typical treatment of partial‐thickness facial burns includes daily washing of the wound with antibacterial soap and water, followed by the application of topical antibiotics (ie, bacitracin).^30^ In a comparison study between honey and silver sulfadiazine in burn wounds, the average healing time was significantly less in the honey group, and more wounds developed complete healing.^31^ All wounds in the honey group were sterile within 7 days, while only 36.5% of the silver sulfadiazine treated wounds became sterile by day 21, reiterating the pro‐healing and antimicrobial effects.^31^ MGH is also effective in wounds colonized with methicillin‐resistant Staphylococcus aureus (MRSA).^32^ The use of MGH in neonatal wounds, which typically are difficult to treat, demonstrated MGH as an effective alternative therapy and prevented amputation of a neonate's toes.^33^

In all the presented cases, the treatment with MGH was received very well, and the application of MGH did not cause any pain or discomfort. Bandages did not stick to the granulation tissue of the wound, and wounds did not reopen upon changing of the MGH and dressings. Moreover, MGH is sterilized using gamma irradiation to eliminate spores of Clostridium, and thus there is no risk on infant botulism, and it can safely be used for pediatric patients.^34^, ^35^, ^36^ Others also support the safe use of MGH for wound healing in children.^33^, ^34^, ^37^, ^38^

Although the number of cases presented is limited, MGH clearly was effective to treat the presented severe wounds in pediatric patients. Based on our own experience, the wounds had healed more rapidly than would have been expected when omitting MGH and using standard therapies instead. This feeling is strongly supported by the burn patient in case 5, of which the healed wound on the leg treated with MGH was used as a skin graft to treat the burn on the back that did not receive MGH treatment. More research to further prove the superiority of MGH is necessary. Despite that each wound and patient is unique, the best option to investigate this is in a double‐blind, randomized MGH/ standard of care‐controlled trial.

## 6 CONCLUSION

Our case series support the safe use of MGH in young pediatric patients for severe wounds of different causes, including an extravasation‐induced injury, a hemangioma, a coccyx ulcer, and thermal burns. MGH was easy to apply and prevented possible infections by covering the wound and via its antimicrobial activity. MGH keeps the wound moisturized while the strong osmotic effect also enhances autolytic debridement, together facilitating wound healing. MGH forms an attractive, cost‐effective approach to treat different wound types in pediatric patients, including severe wounds.

## CONFLICT OF INTEREST

ES is an independent pediatric surgeon and declares no conflict of interest. ES is responsible for the design of the study, the treatments of the patients, and the presentation of the results. NC is employed by Triticum. Triticum provided the L‐Mesitran ointment used in this study free of charge. No other conflict of interest applies. NC was not involved in the design, treatment, and the presentation of the results.

## AUTHOR CONTRIBUTIONS

ES: served responsible for the use of the MGH and considered MGH to be the best option for the treatment in the presented cases. ES: performed the clinical treatments and collected all the data. ES: wrote the results section and made major attributions to the rest of the manuscript. NC: provided the MGH product free of charge and helped writing the rationale and the underlying mechanisms of the MGH product. ES and NC: wrote the discussion of the paper pointing out the specific effects of MGH as observed in the presented cases.
